# The identification of differentially expressed genes between extremes of placental efficiency in maternal line gilts on day 95 of gestation

**DOI:** 10.1186/s12864-019-5626-0

**Published:** 2019-03-29

**Authors:** Shanice K. Krombeen, Vijay Shankar, Rooksana E. Noorai, Christopher A. Saski, Julia L. Sharp, Matthew E. Wilson, Tiffany A. Wilmoth

**Affiliations:** 1 Department of Animal and Veterinary Science, Clemson University, Clemson, SC 29634 USA; 2 Center for Human Genetics, Clemson University, Greenwood, SC 29646 USA; 3 Genomics and Bioinformatics Facility, Clemson University, Clemson, SC 29634 USA; 4 Department of Plant and Environmental Sciences, Clemson University, Clemson, SC 29634 USA; 5 Department of Statistics, Colorado State University, Fort Collins, CO 80523 USA; 6 Division of Animal and Nutritional Sciences, West Virginia University, Morgantown, WV 26506 USA

**Keywords:** Endometrium, Fetal weight, Gene expression, Pigs, Placenta, Placental efficiency, Placental weight

## Abstract

**Background:**

Placental efficiency (PE) describes the relationship between placental and fetal weights (fetal wt/placental wt). Within litters, PE can vary drastically, resulting in similarly sized pigs associated with differently sized placentas, up to a 25% weight difference. However, the mechanisms enabling the smaller placenta to grow a comparable littermate are unknown. To elucidate potential mechanisms, morphological measurements and gene expression profiles in placental and associated endometrial tissues of high PE and low PE feto-placental units were compared. Tissue samples were obtained from eight maternal line gilts during gestational day 95 ovario-hysterectomies. RNA was extracted from tissues of feto-placental units with the highest and lowest PE in each litter and sequenced.

**Results:**

Morphological measurements, except placental weight, were not different (*P* > 0.05) between high and low PE. No DEG were identified in the endometrium and 214 DEG were identified in the placenta (FDR < 0.1), of which 48% were upregulated and 52% were downregulated. Gene ontology (GO) analysis revealed that a large percentage of DEG were involved in catalytic activity, binding, transporter activity, metabolism, biological regulation, and localization. Four GO terms were enriched in the upregulated genes and no terms were enriched in the downregulated genes (FDR < 0.05). Eight statistically significant correlations (*P* < 0.05) were identified between the morphological measurements and DEG.

**Conclusion:**

Morphological measures between high and low PE verified comparisons were of similarly sized pigs grown on different sized placentas, and indicated that any negative effects of a reduced placental size on fetal growth were not evident by day 95. The identification of DEG in the placenta, but absence of DEG in the endometrium confirmed that the placenta responds to the fetus. The GO analyses provided evidence that extremes of PE are differentially regulated, affecting components of placental transport capacity like nutrient transport and blood flow. However, alternative GO terms were identified, indicating the complexity of the relationship between placental and fetal weights. These findings support the use of PE as a marker of placental function and provide novel insight into the genetic control of PE, but further research is required to make PE production applicable.

## Background

Placental efficiency (PE), quantified by the ratio of fetal or birth weight to placental weight, is commonly used as a marker of placental function in humans and animals. The ratio reflects grams of fetus produced per gram of placenta [1]. In general, it is assumed that high PE values associated with averaged sized fetuses represent placentas with a greater nutrient transport capacity, while low PE values associated with growth restricted fetuses represent placentas with a reduced nutrient transport capacity or a failure to adapt.

In humans, PE (birth wt/placental wt) is reduced during pregnancy complications like fetal growth restriction, small for gestational age, gestational diabetes mellitus, and pre-eclampsia [2]. Therefore, the ratio is often used to predict abnormal fetal growth, and consequently health later in life. However, whether alterations in PE truly reflect adaptations in human placental nutrient transport capacity remains unclear. The most compelling evidence for an association between the two is in mice, with conflicting reports in humans [2]. Coan and others [3] evaluated placental nutrient transport capacity in mice with natural variations in placental size to determine if the smallest placenta in a litter of appropriately grown fetuses was the most efficient. The authors reported near term fetuses with lighter placentas were of comparable weight to fetuses with heavier placentas, and thus, PE was greater in the lightest placentas. Additionally, expression of *Slc2a1*, a glucose transporter gene, and *Slc38a2*, an amino acid transporter gene, were upregulated in the lightest placentas, providing evidence that high PE placentas adapt to meet the nutrient demands of the growing fetus.

Natural variations in PE are also apparent in pigs, a litter bearing species. These variations are not only between, but also within, breeds and even within litters [4]. Within a litter, PE can vary drastically, resulting in similarly sized pigs grown on very different placentas, with up to a 25% weight difference [5]. High PE placenta are smaller in size than low PE placenta, thus, high PE placentas occupy less space in the uterus and still grow an averaged sized littermate. While the use of PE as a selection tool to increase litter size has been debated [6], PE may provide an opportunity to optimize reproductive performance. The average litter size of U.S. production breeds has continued to increase over time and is currently 10.6 [7], but ovulation rates [8] and teat numbers [9] indicate the maximum has not been reached. At the same time, increases in litter size have resulted in lower birth weights, less uniform litters, and greater preweaning mortalities, minimizing the benefits of producing more pigs per litter. Increasing PE in these larger litters may normalize birth weights and, as a result, increase preweaning survival. In fact, Vernunft and others [10] reported on the relationship between placental size and measures of reproductive performance in modern Landrace sows. The authors concluded sows with larger litters and shorter placentas can rescue placental function. However, the compensatory mechanisms driving the growth of an adequately sized fetus on a smaller placenta are still being investigated.

In comparisons of breeds differing in PE, the increased efficiency of high PE placentas was attributed to greater vascularity [11]. Yet, variations in PE within production breed litters on day 90 of gestation could not be attributed to differences in vascular density (VD) despite increased expression of vascular endothelial growth factor and associated receptors in high PE placentas [12]. Recently, Krombeen and others [5] reported placental VD was positively related to PE on day 110 of gestation in maternal line gilts. The results of Vonnahme and Ford [12] in conjunction with Krombeen and others [5] suggest morphological adaptations, like increases in VD, may occur later in gestation (day 90 to term) to maintain fetal growth when placental size is reduced.

Krombeen and others [5] also investigated the relationship between PE and seven genes encoding glucose, amino acid, or fatty acid transporters in the placenta and adjacent endometrium of maternal line gilts on day 70, 90, and 110 of gestation. Based on conditional effects plots, variations in PE on day 70 of gestation were related to alterations in amino acid transporter expression (*SLC7A7*, S*LC7A1*, *SLC3A1*) in the placenta and endometrium. On day 90 of gestation, PE was positively related to placental expression of a glucose transporter (*SLC2A3*) and negatively related to endometrial expression of two cationic amino acid transporters (*SLC7A1* and *SLC7A2*) and a very long chain fatty acid transporter (*SLC27A1*). Near term (day 110), PE was negatively related to two amino acid transporters (*SLC7A7* and *SLC7A1*) and a glucose transporter (*SLC2A3*). The results of Krombeen and others [5] agree with those of Coan and others [3] and supports the use of PE as a marker of placental function.

Although the study by Krombeen and others [5] provides evidence for an association between PE and placental nutrient transport capacity, only seven genes encoding nutrient transporters were investigated. Zhou and others [13] detected 226 and 577 differentially expressed genes on gestational days 75 and 90, respectively, between two breeds with differing PE. Similarly, Kwon and others [14] identified 588 differentially expressed genes in placentas from larger litter sizes compared to smaller litter sizes. Therefore, it is likely the compensatory mechanisms enabling comparable fetal growth despite reductions in placental size (high PE) are controlled by many genes and the interactions of those genes, as seen in Zhou and others [13] and Kwon and others [14].

However, the global expression of genes in the placenta of the high and low PE feto-placental units within a litter has not been investigated. The hypothesis of this work was that extremes of PE would be associated with differentially expressed genes (DEG) that affect fetal growth via gene products that promote growth, vascularity, and/or nutrient transport. The main objective was to determine and understand the role of gene expression profiles in placental and associated endometrial tissues of high PE and low PE feto-placental units. A secondary objective was to compare fetal and utero-placental measurements of high PE and low PE units.

## Results

### Fetal and utero-placental measurements

**Fig. 1:**
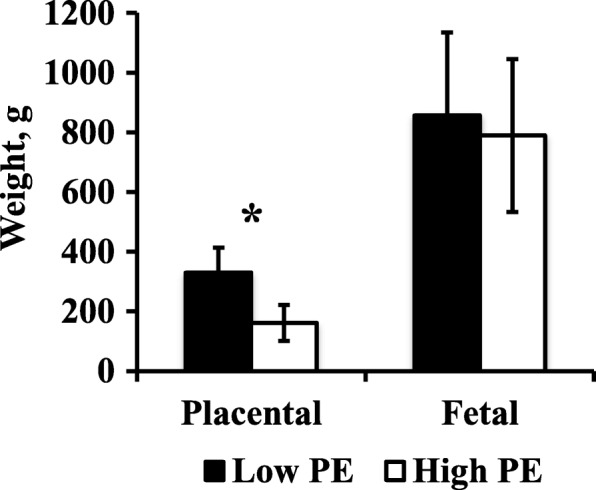
Mean placental and fetal weight. Mean placental weight and fetal weight of low PE and high PE feto-placental units on day 95 of gestation in pigs. Data presented as means ± SD. Asterisk (*) indicates *P* < 0.05

**Fig. 2:**
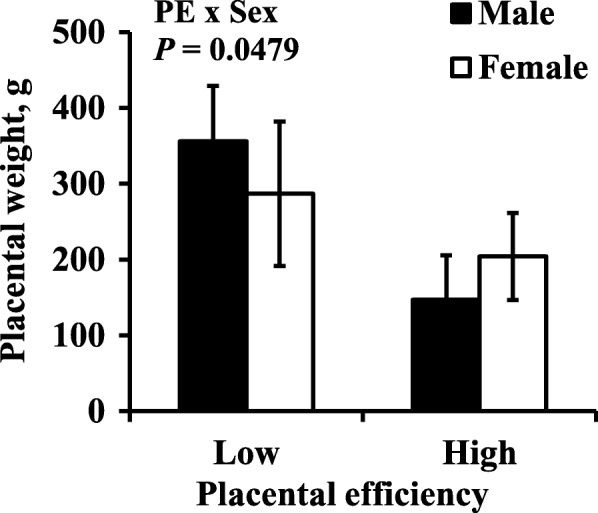
Mean placental weight by sex. Mean placental weight in low PE and high PE male and female feto-placental units on day 95 of gestation. Data presented as means ± SD

Mean litter size was 10.75 and ranged from 5 to 15. There was no association between litter size and PE (*r* = − 0.04, *P* = 0.72). Table 1 contains least square means ± SE of fetal and placental measurements. Mean placental weight was lower in the high PE group compared to the low PE group (*P* = 0.0002, Fig. 1), but mean fetal weight was not different between high and low PE (*P* = 0.5914, Fig. 1). While the effect of sex was not significant for placental weight or fetal weight, there was a significant interaction between PE and sex for placental weight (*P* = 0.0479, Table 1). Males had lower weight placentas than females in the high PE group, but the opposite was true in the low PE group (Fig. 2).

**Table 1:** Least square means ± SE of fetal and utero-placental measurements of high PE and low PE units

Measurement^b^	Placental efficiency		*P*-value^a^		
	Low	High	PE	Sex	PE × Sex
Placental wt, g	322.21 ± 24.14	172.37 ± 25.39	0.0002*	0.7257	0.0479*
Fetal wt, g	831.28 ± 99.36	782.04 ± 106.59	0.5914	0.3647	0.4386
ISL, cm	21.08 ± 3.05	22.19 ± 3.60	0.8208	0.6657	0.9710
CRL, cm	25.88 ± 1.35	25.02 ± 1.42	0.4338	0.2303	0.7022
Girth, cm	17.58 ± 1.34	18.27 ± 2.02	0.7310	0.9900	0.4043
Heart wt, g	7.63 ± 1.06	5.92 ± 1.19	0.2071	0.7930	0.3736
Liver wt, g	21.97 ± 3.14	17.86 ± 3.38	0.1925	0.4927	0.5075
Brain wt, g	19.95 ± 0.72	19.73 ± 0.83	0.8254	0.5937	0.7535
ST wt, g	1.60 ± 0.22	1.50 ± 0.23	0.6886	0.6552	0.8463
Placental VD, %	8.19 ± 1.35	5.74 ± 1.56	0.2689	0.3511	0.4941
Endometrial VD, %	6.15 ± 1.73	7.48 ± 1.96	0.5463	0.8872	0.4179

^a^Effect of placental efficiency (PE), effect of sex (Sex), effect of placental efficiency by sex interaction (PE × Sex). ^b^*ISL* Implantation site length, *CRL* crown-rump length, *ST* semitendinosus, *VD* vascular density. (*) asterisk indicates *P* < 0.05

Mean implantation site length (ISL), crown-rump length (CRL), girth, heart weight, liver weight, brain weight, and semitendinosus (ST) weight were not significantly different between the high PE group and the low PE group (*P* = 0.8208, 0.4338, 0.7310, 0.2071, 0.1925, 0.8254, and 0.6886, respectively). There were also no significant differences in mean placental or endometrial vascular density (VD) between high PE and low PE (*P* = 0.2689, 0.5463, respectively). For these variables, there was no significant effect of sex and there were no significant interactions between PE and sex.

### Differential gene expression and gene ontology

**Fig. 3:**
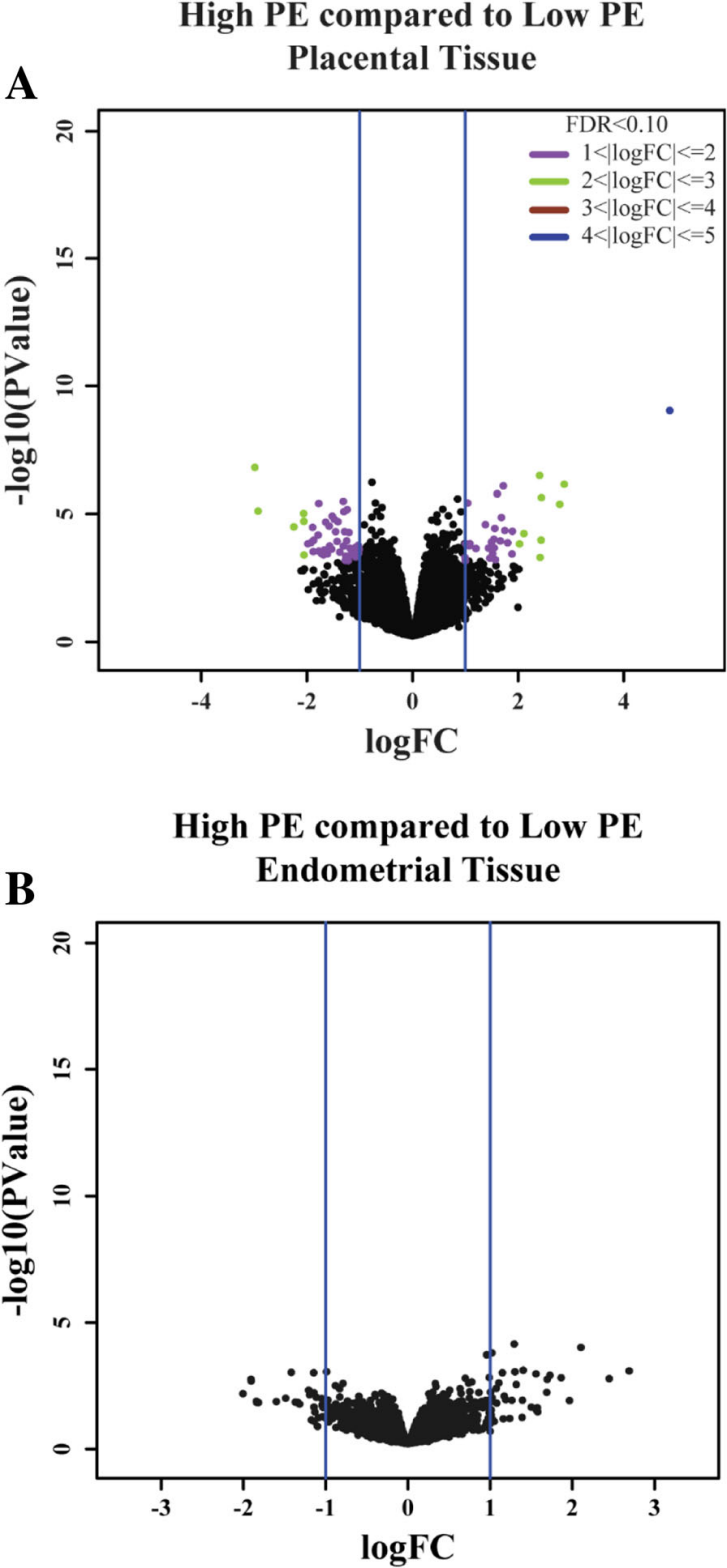
Gene Expression. **a** Volcano plot of DEG in high PE compared to low PE placental tissue on day 95 of gestation in pigs. Black dots indicate FDR > 0.10 or |log_2_FC| < 1. Non-black dots indicate DEG (FDR < 0.10, |log_2_FC| ≥ 1). Dot colors indicate log_2_FC range. **b** Volcano plot of DEG in high PE compared to low PE endometrial tissues on day 95 of gestation in pigs. Black dots indicate FDR > 0.10 or |log_2_FC| < 1

The number of genes expressed in the placenta and endometrium was 20,280. In total, 214 DEG (FDR < 0.1) were identified in the placenta (Fig. 3a) and 0 DEG (FDR < 0.1) were identified in the endometrium (Fig. 3b). Of the DEG in the placenta, 103 genes were upregulated (33 log fold change (log_2_FC) ≥ 1; 70 0 < log_2_FC < 1.0) and 111 genes were downregulated (49 log_2_FC ≤ − 1.0; 62 -1.0 < log_2_FC < 0). Table 2 lists a subset of the candidate genes in high PE compared to low PE placentas.

**Table 2:** Candidate genes in high PE compared to low PE placentas

Gene Symbol	Gene Name	log_2_FC^a^	FDR *P*-value^2^	Gene Ontology (MF; BP)^b^
SAA2	Serum Amyloid A2	4.89	0.00002	binding, molecular transducer activity; locomotion
SPHKAP	Sphingosine Kinase Type 1	2.88	0.00388	binding
DKK1	Dickkopf-1	2.80	0.00876	binding, molecular transducer activity, molecular function regulator; biological regulation
CYP4F22	Cytochrome P450 Family 4 Subfamily F Member 22	2.44	0.05077	catalytic activity; metabolic process
FBP2	Fructose-1, 6-Bisphosphatase Isoenzyme 2	2.42	0.08290	catalytic activity; metabolic process
PCDHB1	Protocadherin Beta-1 Isoform X2	2.42	0.00302	;biological adhesion
SLC45A3	Solute Carrier Family 45 Member 3	1.89	0.07267	transporter activity, metabolic process
ASIC1	Acid Sensing Ion Channel 1 Isoform X2	1.81	0.05118	transporter activity; biological regulation, localization, multicellular organismal process
CELA1	Chymotrypsin-like Elastase Family Member 1	1.55	0.04841	catalytic activity
LEP	Leptin	1.01	0.05194	growth factor activity, hormone activity^c^
ATP13A3	Probable Cation-Transporting ATPase 13A3	0.82	0.06425	; biological regulation
SLC4A7	Solute Carrier Family 4 Member 7	0.77	0.04207	transporter activity
CTSH	Pro-cathepsin H	0.71	0.07854	; positive regulation of angiogenesis^c^
SLC52A3	Solute Carrier Family 52 Member 3	0.68	0.01438	molecular transducer activity; biological regulation
SLC23A2	Solute Carrier Family 23 Member 2	0.59	0.06802	transporter activity; localization
AGR2	Anterior Gradient Protein 2 Homolog	−2.99	0.00045	; biological regulation, cellular process
EMB	Embigin	−2.06	0.01288	binding; biological adhesion, cellular process
STEAP1	Six-Transmembrane Epithelial Antigen of Prostate 1	−1.98	0.05194	catalytic activity; localization
SARDH	Sarcosine Dehydrogenase	−1.89	0.02504	catalytic activity; metabolic process
STEAP2	Six Transmembrane Epithelial Antigen of the Prostate 2	−1.72	0.07267	catalytic activity; localization
MRP4	Multidrug Resistance-Associated Protein 4-like	−1.61	0.07267	transporter activity
KCNJ2	Potassium Channel, Inwardly Rectifying Subfamily J, Member 2	−1.31	0.00875	transporter activity
ANGPT1	Angiopoietin-1	−0.75	0.07031	binding; biological regulation, biological adhesion

^a^*log*_*2*_*FC* log_2_ fold change. ^2^*FDR P-value* False discovery rate adjusted *P*-value, level of significance *P* < 0.10. ^b^*MF* Molecular function, *BP* biological process. Genes above dotted line were among ten most upregulated or downregulated. ^c^No hits for Panther GO slim terms, specific GO terms used instead

**Fig. 4:**
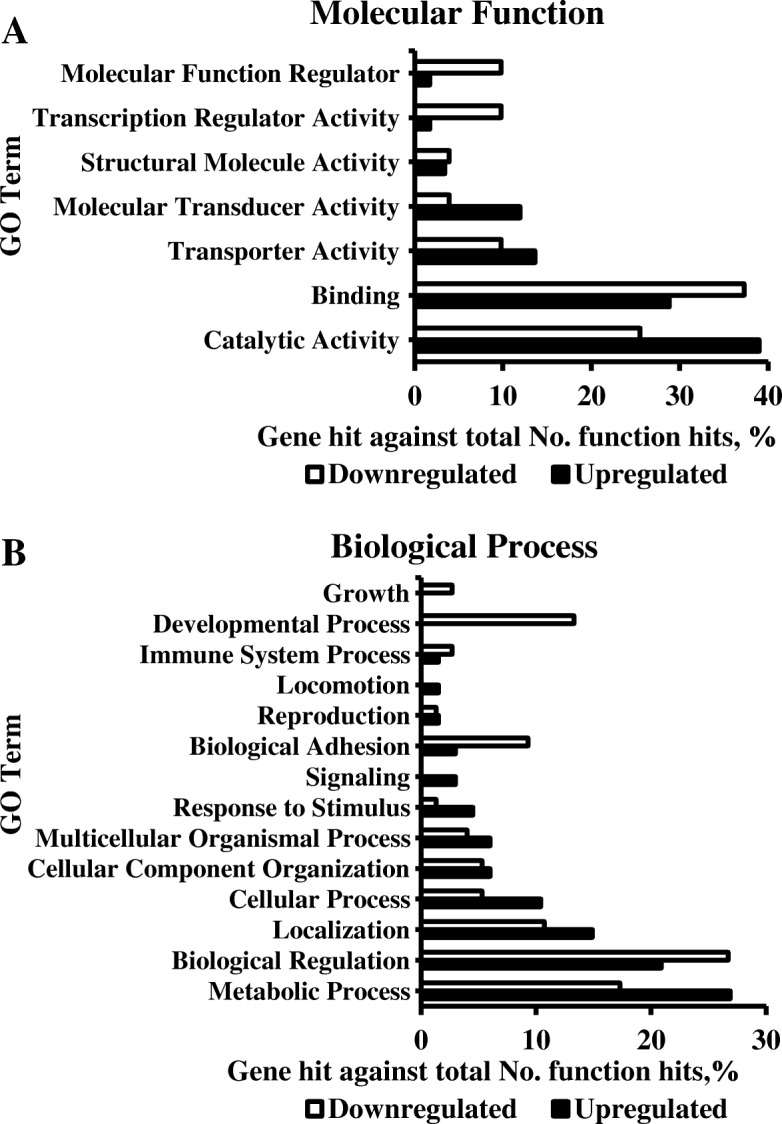
Gene Ontology Functional Classification Analysis. **a** Molecular function GO terms of DEG in high PE compared to low PE placentas on day 95 of gestation in pigs. **b** Biological Process GO terms of DEG in high PE compared to low PE placentas on day 95 of gestation in pigs. Subfigures included 160 of the 214 DEG

The results of the functional classification analysis are presented in Fig. 4, which illustrates percentages of upregulated compared to downregulated genes designated by MF (Fig. 4a) or BP (Fig. 4b) gene ontology (GO) terms for high PE compared to low PE. The MF of catalytic activity, binding, and transporter activity had the greatest representation. More catalytic activity genes (39% vs 25.5%) and transporter activity genes (14% vs 10%) were upregulated than downregulated, while the opposite was true for binding activity genes (29% vs 37%). Additionally, a greater percentage of downregulated genes compared to upregulated genes were molecular function regulators or had transcription regulator activity (both 2% vs 10%). The BP terms with the greatest representation were metabolic process, biological regulation, and localization. Upregulated genes were involved in metabolic processes (27% vs 17%) and localization (15% vs 11%) more often than downregulated genes, while downregulated genes were involved in biological regulation more often than upregulated genes (21% vs 27%). Also, the terms developmental process (0% vs 13%) and biological adhesion (3 vs 9%) were associated with more downregulated than upregulated genes.

Table 3 contains the results of the GO enrichment analysis performed on the DEG in high PE compared low PE placentas. Four terms were significantly (FDR < 0.05) enriched in the upregulated genes and no terms were significantly enriched in the downregulated genes. The MF terms sodium-dependent multivitamin transmembrane transporter activity and nucleobase transmembrane transporter activity, the BP term nucleobase transport, and the cellular component term extracellular exosome were enriched.

**Table 3:** Gene Ontology Enrichment Analysis

Ontology^a^	Category	GO Term	No. DEG in Category	No. in Category	FDR *P*-value	Gene Symbol^b^
MF	GO:0008523	Sodium-dependent multivitamin transmembrane transporter activity	2	3	0.0486	*SLC23A2, LOC102159690*
MF	GO:0015205	Nucleobase transmembrane transporter activity	2	3	0.0486	*SLC23A2, LOC102159690*
BP	GO:0015851	Nucleobase transport	2	3	0.0486	*SLC23A2, LOC102159690*
CC	GO:0070062	Extracellular exosome	20	2283	0.0486	*TXN, TXNDC8, MGST3, CADM4, PI16, CTSH, ABCB1, FBP2, RPL15, ITIH3, PDHB, PEBP1, PPA1, GCA, HSPE1, SPHKAP, EFHD1, ENTPD6, AK2, GLA*

Significantly enriched GO terms of upregulated genes in high PE compared to low PE placentas on day 95 of gestation in pigs. Level of significance *P* < 0.05. ^a^*MF* Molecular function*, BP* Biological process*, CC* Cellular component*, GO* Gene ontology*, DEG* Differentially expressed genes, *FDR P-value* False discovery rate adjusted *P*-value, ^b^*SLC23A2* Solute carrier family 23 member 2, *LOC102159690* solute carrier family 23 member 2-like, *TXN* Thioredoxin*, TXNDC8* Thioredoxin domain-containing protein 8*, MGST3* Microsomal glutathione S-transferase 3*, CADM4* Cell adhesion molecule 4*, PI16* Peptidase inhibitor 16 precursor*, CTSH* Pro-cathepsin H*, ABCB1* ATP-binding cassette subfamily B member 1 isoform X2*, FBP2* Fructose-1, 6-biphosphatase isoenzyme 2*, RPL15* Ribosomal protein L15*, ITIH3* Inter-alpha-trypsin inhibitor heavy chain H3*, PDHB* Pyruvate dehydrogenase E1 component subunit beta*, PEBP1* Phosphatidylethanolamine-binding protein 1*, PPA1* Inorganic pyrophosphatase*, GCA* Grancalcin*, HSPE1* 10 KDa heat shock protein, mitochondrial*, SPHKAP* A-kinase anchoring protein*, EFHD1* EF-hand domain-containing protein D1 isoform X2*, ENTPD6* Ectonucleoside triphosphate diphosphohydrolase 6*, AK2* Adenylate kinase 2*, GLA* Alpha-galactosidase A

### Fetal/utero-placental measurements and differentially expressed genes

Eight significant correlations (*P* < 0.05) were identified between fetal and utero-placental measurements, and the 214 DEG in high PE compared to low PE placentas (Table 4), of which 5 were placental weight and 3 were PE correlations. Placental weight was positively correlated with GRINL1A complex locus 1 (GCOM1, *r* = 0.82, *P* = 0.0214), gene19656 (LOC100739517, *r* = 0.78, *P* = 0.0257), TOX high mobility group box family member 3 (TOX3, *r* = 0.77, *P* = 0.0257), and ATP-binding cassette sub-family G member 2 (ABCG2, *r* = 0.76, *P* = 0.0257), but negatively correlated with ras-related protein rab-6B (RAB6B, *r* = − 0.76, *P* = 0.0257). Placental efficiency was positively correlated with gene12188 (LOC100156118, *r* = 0.81, *P* = 0.0214), transmembrane protein 199 (TMEM199, *r* = 0.81, *P* = 0.0214), and proto-cadherin beta 1 (PCDHB1, *r* = 0.76, *P* = 0.0428).

**Table 4:** Significant correlations between fetal/utero-placental measurements and DEG in high PE compared to low PE placentas

Correlation Data^a^			DEG Data^b^				
Measurement* Gene	Correlation	FDR *P*-value	Gene Symbol	Protein	Molecular Function	log FC	FDR *P*-value
PW*gene1223	0.82	0.0214	*GCOM1*	Myocardial Zonula Adherens Protein	N/A	−0.43	0.0769
PE*gene12188	0.81	0.0214	LOC100156118	N/A	N/A	0.63	0.0484
PE*gene24847	0.81	0.0214	*TMEM199*	Transmembrane Protein 199	N/A	0.29	0.0421
PW*gene19656	0.78	0.0257	LOC100739517	ATP-Binding Cassette Sub-Family G Member 2	N/A	−0.61	0.0144
PW*gene13353	0.77	0.0257	*TOX3*	TOX High Mobility Group Box Family Member 3	Chromatin binding, Phosphoprotein binding, Protein homodimerization activity, Estrogen response element binding	−1.41	0.0183
PW*gene19659	0.76	0.0257	*ABCG2*	ATP-Binding Cassette Sub-Family G Member 2	ATP binding, ATPase activity coupled to transmembrane movement of substance, Cholesterol transporter activity	−0.77	0.0039
PE*gene6367	0.76	0.0428	*PCDHB1*	Protocadherin Beta-1 Isoform X2	Calcium ion binding	2.42	0.0030
PW*gene26368	−0.76	0.0257	*RAB6B*	Ras-Related Protein Rab-6B	GTP binding, GTPase activity, Myosin V binding	0.86	0.0084

^a^*PW* Placental weight, *PE* placental efficiency, *FDR P-value* false discovery rate adjusted *P*-value, level of significance *P* < 0.05. ^b^*DEG* Differentially expressed genes*, N/A* not available*, log*_*2*_*FC* log_2_ fold change*, FDR P-value* false discovery rate adjusted *P*-value, level of significance *P* < 0.10

## Discussion

### Fetal and utero-placental measurements

Placental weight was lower in the high PE group compared to the low PE group, but fetal weight was not different between high and low PE. These results agree with Krombeen and others [5] and confirm high PE placentas are smaller than low PE placentas, yet each grow a littermate of comparable size. There was a significant interaction between PE and sex for placental weight, which to the authors’ knowledge has not previously been reported in PE studies.

The lack of a difference in ISL between high PE and low PE was unexpected as the basis for high PE is a smaller but more efficient placenta that occupies less room within the uterus [4, 11]. Furthermore, a previous study reported high PE ISL were shorter than low PE ISL [12]. The conflicting results suggest ISL differs when PE is used as a selection tool as opposed to observing natural variations in PE. Alternatively, the folded placental trophoblast-endometrial epithelial bilayer width may be greater in feto-placental units with reduced placental size and comparable fetal growth (high PE). Vallet and Freking [15], reported greater fold widths were associated with the smallest pig fetuses in a litter and may increase PE via a larger surface area for exchange between the maternal and fetal circulations.

In addition to ISL, CRL, girth, heart weight, liver weight, brain weight, and ST weight were not significantly different between the high PE group and the low PE group. Crown-rump length and girth are highly correlated to fetal age [16] and weight [17, 18], and can be used to predict neonatal survival [19] and post-natal growth performance [20]. Considering these fetal measurements did not differ based on PE, the survival and postnatal growth performance of pigs grown on high PE placentas should not be negatively affected. Moreover, the absence of significant differences in fetal organ and tissue weights supports data from Krombeen and others [5], indicating any negative effects of a reduced placental size on fetal growth are not evident by term.

Placental VD and endometrial VD were also not significantly different between high PE and low PE. High PE in more prolific breeds has been attributed to increased placental VD during late gestation [11]; however, the role VD plays in extremes of PE within production breeds is less clear. Vonnahme and Ford [12] reported there was no additional increase in placental or endometrial VD to account for the increased efficiency of high PE placentas on day 90 of gestation in Yorkshires, despite increased expression of a vascular growth factor and its associated receptors. Conversely, Krombeen and others [5] identified a positive relationship between placental VD and PE on day 110 of gestation in maternal line gilts. It is conceivable that increased vascular permeability or reduced placental-endometrial intercapillary distance contribute to high PE, as suggested by Vonnahme and Ford [12], and/or changes in VD occur after day 90, as suggested by Krombeen and others [5].

### Differential gene expression and gene ontology

A total of 214 DEG were identified in the placenta and 0 DEG were identified in the endometrium. Since the placenta is conceptus derived, it is not surprising that a greater number of genes would be differentially expressed in the placenta than in the endometrium, which is maternal tissue. Of the genes expressed in the placenta, only 214 were differentially expressed or 1.06% of the transcriptome. The small percentage of DEG identified in this study could be attributed to the comparison of two naturally occurring states as opposed to two treatment groups, the gestational day evaluated, and/or the expression level measured.

Nonetheless, the functional classification analysis performed on the DEG in high PE compared to low PE placentas identified molecular functions (MF) and biological processes (BP) associated with the phenotype. The MF of catalytic activity, binding, and transporter activity had the greatest representation. Catalytic activity was a MF of more upregulated than downregulated genes.

Upregulated candidate genes with catalytic activity included cytochrome P450 family 4 subfamily F member 22 (*CYP4F22*), fructose-1, 6-bisphosphatase isoenzyme 2 (*FBP2*), and chymotrypsin-like elastase family member 1 (*CELA1*). The catalytic activity of the products of *CYP4F22*, recently identified as an ultra-long chain fatty acid omega hydroxylase [21], and *FBP2*, encoding the gluconeogenic enzyme fructose-1,-6,biphosphatase-2 [22], suggests the metabolic state of high PE and low PE placentas differs. The gene *CELA1* encodes for an enzyme that degrades the protein elastin, a component of the vascular matrix. Data in mice indicate there is a positive association between *CELA1* and angiogenesis [23, 24]. While the catalytic activity of *CELA1* may have a role in vascularity, no differences in VD were detected between high PE and low PE placentas.

Catalytic activity was also a function of downregulated genes; six transmembrane epithelial antigen of prostate 1 (*STEAP1)*, six transmembrane epithelial antigen prostate 2 (*STEAP2*), and sarcosine dehydrogenase (*SARDH)* were candidate genes with catalytic activity. The STEAP family of proteins function as metal reductases, enabling the transport of iron and copper across the plasma membrane, and superoxide synthases, generating superoxide [25, 26]. The gene *SARDH* encodes for a mitochondrial enzyme that catalyzes the conversion of sarcosine to glycine, a major amino acid involved in an array of BP [27]. The downregulation of these genes in high PE compared to low PE suggests metabolism differs by PE.

The MF GO term with the second greatest representation in the DEG was binding, with fewer upregulated than downregulated genes involved in binding. Serum amyloid A2 (*SAA2*), sphingosine kinase type 1 interacting protein (*SPHKAP*), and dickkopf-1 (DKK1), were the three most upregulated genes in high PE compared to low PE placentas and shared the MF of binding. These genes encode a major acute phase protein involved in the innate immune response [28], an A-kinase anchoring protein involved in second messenger intracellular signaling [29], and a glycoprotein that is an inhibitor of the Wnt signaling pathway [30], respectively. In general, it appears the binding activity of these gene products is relevant to cell signaling pathways. While the role of these genes products within the placenta requires further investigation, SAA2 and DKK1 have been implemented in lipid metabolism [31, 32] and angiogenesis [33–35].

Embigin (*EMB*) and angiopoietin 1 (*ANGPT1*) were downregulated candidate genes with the MF binding. The gene product of *EMB* is a transmembrane glycoprotein required for the localization and function of MCT2, a plasma membrane transporter of pyruvate, lactate, and ketone bodies [36]. The downregulation of *EMB* in high PE placentas suggests a lack of transport of these substrates, but given that pyruvate and lactate are gluconeogenic precursors and *FBP2*, encoding a gluconeogenic enzyme, was upregulated, it is plausible that these substrates may be metabolized to produce glucose in high PE placentas. On the other hand, *ANGPT1* belongs to a family of endothelial growth factors and is a glycoprotein that inhibits endothelial permeability [37]. Thus, the downregulation of *ANGPT1* in high PE compared to low PE placentas indicates vascular permeability may be increased in high PE placentas.

Transporter activity was another MF of the DEG, with a greater percentage of upregulated than downregulated genes involved in transporter activity. The following are candidate genes for PE with transporter activity: solute carrier family 45 member 3 (*SLC45A3*), acid sensing ion channel subunit 1 (*ASIC1*), solute carrier family 4 member 7 *(SLC4A7*), and solute carrier family 23 member 2 (*SLC23A2*). The gene *SLC45A3* encodes for a novel H+ sucrose symporter, suggested to also transport fructose and glucose [38, 39]. Verification of this function in the pig placenta is required, but it appears sugar transport is increased in high PE placentas. Acid sensing ion channel 1 isoform X2 (*ASIC1*) encodes for a proton-gated sodium ion channel that localizes to the plasma membrane and golgi apparatus. Expression is most common in neurons [40] and to the authors’ knowledge has not been reported in the pig placenta. Assuming a similar MF, upregulation of *ASIC1* in high PE placentas indicates sodium ion transmembrane transport differs based on PE. The gene *SLC4A7* encodes for a sodium bicarbonate (Na^+^: HCO_3_^−^) cotransporter [41] and the gene *SLC23A2* encodes for a sodium dependent ascorbate (vitamin C) co-transporter (2Na^+^: ascorbate) [42]. In rodent models, both transporters have been implemented in the control of vasodilation [41, 43]. Thus, the upregulation of these transporters in high PE placentas may alter placental vascular tone, but further research is required to elucidate the role of these transporters in the pig placenta.

Transporter activity was also a function of downregulated genes, like multidrug resistance associated protein 4 (*MRP4*) and potassium channel inwardly rectifying subfamily J member 2 (*KCNJ2*). The gene *MRP4* encodes for an active transporter protein with a broad substrate specificity [44]. Interpretation of the significance of *MRP4* downregulation in high PE placentas requires further research into the substrates of MRP4 in the pig placenta. The gene *KCNJ2* encodes the inwardly rectifying potassium channel K_IR_2.1. Expression of K_IR_2.1 has been reported in the human placenta, but the tissue specific function is unknown [45, 46]. Disruption of the potassium channel in mice indicated K_IR_2.1 mediates vasodilation [47]. The downregulation of KCNJ2 in high PE placentas suggest potassium transport and potentially vasodilation may be altered by PE.

Interestingly, two MF terms, molecular function regulator and transcription regulator activity, were functions of mostly downregulated genes. Molecular function regulators modulate a gene products activity and are often enzyme regulators or channel regulators [48]. Downregulated genes associated with this term were mostly enzyme regulators modulating intracellular activity. Transcription regulator activity describes the function of controlling gene expression at the level of transcription [48]. Accordingly, downregulated genes with this function encoded for transcription factors. Thus, downregulated genes were involved in the control of gene expression and the activity of gene products, which is not surprising given that this study compares high PE to low PE placentas.

Functional classification of the DEG also identified BP associated with the phenotype. Metabolic process, biological regulation, and localization had the greatest representation among the DEG. The gene products of more upregulated than downregulated genes were involved in metabolic processes. This is as expected given the MF catalytic activity and transporter activity had the greatest representation among the upregulated genes, and these functions are often involved in metabolism. For instance, *CYP4F22* encodes for an ultra-long chain fatty acid omega hydroxylase, an enzyme of fatty acid metabolism [21]. Similarly, the genes *FBP2* and *SLC45A3* encode for a gluconeogenic enzyme [22] and sucrose transporter [38, 39], respectively, both of which are involved in carbohydrate metabolism. Furthermore, the identification of metabolic processes as the BP with the greatest representation in the DEG indicates extremes of PE are related to metabolism.

Biological regulation was also a BP term of a large percentage of the DEG and is a broad term encompassing genes products that modulate part of a BP [48]. More downregulated than upregulated genes were involved in biological regulation. Biological regulation was a term of anterior gradient protein 2 (*AGR2*), the most downregulated gene in high PE compared to low PE placentas. The gene *AGR2* encodes for a member of the protein disulfide isomerase family of endoplasmic reticulum proteins, which are essential to post-translational folding [49]. The protein has been implemented in epithelial barrier function and cell proliferation. Moreover, it has been suggested that *AGR2* downregulation in sheep placentomes may serve as an adaptive placental mechanism to support fetal growth during stress by reducing the proliferative actions of AGR2 [50]. Whether *AGR2* regulates a similar BP in the pig placenta remains to be determined. Other downregulated genes involved in biological regulation included *ANGPT1*, a regulator of vascular permeability [37], and several other genes encoding transcription factors.

While there were more downregulated genes involved in biological regulation, a significant percentage of upregulated genes were also biological regulators. Among these were probable cation-transporting ATPase 13A3 (*ATP13A3*) and solute carrier family 52 member 3 (*SLC52A3*). The gene *ATP13A3* encodes a protein involved in calcium ion transmembrane transport, with evidence of polyamine transport in worms [51] and humans [52]. Polyamines perform numerous essential functions in mammalian physiology and are known regulators of placental growth and angiogenesis [53]. Thus, *ATP13A3* may be a candidate gene for high PE, but additional research into the substrate specificity of *ATP13A3* in the pig placenta is required. The gene *SLC52A3* also encodes for a transporter, but with riboflavin (vitamin B2) specificity. Riboflavin is a regulator of metabolism via the active forms flavin mononucleotide and flavin adenine dinucleotide. Knockout of *Slc52a3* in mice caused reduced riboflavin concentrations in pups resulting in death, with signs of hyperlipidemia and hypoglycemia [54]. Thus, the upregulation of *SLC52A3* in high PE placentas likely regulates metabolism. Overall, the large percentage of DEG involved in biological regulation suggests extremes of PE may originate from differential regulation of several BP.

Localization was also a BP term of a large percentage of the DEG, with greater representation in upregulated than downregulated genes. The term describes the transportation or maintenance of a substance to a location [48]. The percentage of DEG involved in localization is reflective of the MF with greatest representation among the DEG (catalytic activity, binding, and transporter activity). For example, *ASIC1* and *SLC23A2* were upregulated and encode for a sodium ion transmembrane transporter [40] and an ascorbate transmembrane co-transporter [42], respectively. Similarly, the downregulated genes *STEAP1* and *STEAP2* encode for transmembrane proteins with metal reductase and superoxide synthase activity [25, 26]. Furthermore, the DEG involved in localization indicate micronutrient transport differs in extremes of PE.

The terms developmental process and biological adhesion were BP of mainly downregulated genes. The term developmental process describes BP involved in the progression of a living unit [48]. Downregulated genes with this BP mostly encoded transcription factors, suggesting extremes of PE are driven by differences in the control of development. Conversely, biological adhesion was a term of downregulated genes involved in cell adhesion. Among these were *EMB* and *ANGPT1*, encoding a transmembrane protein that localizes monocarboxylate transporters to the cell membrane [36] and an endothelial growth factor known to inhibit endothelial permeability [37], respectively. Thus, downregulated genes involved in biological adhesion may affect the nutrient transport capacity of high PE placentas.

A GO enrichment analysis was also performed on the DEG in high PE compared to low PE placentas. Only 4 enriched terms were identified, which was probably due to the limited number of DEG in the input list. The MF terms sodium-dependent multivitamin transmembrane transporter activity and nucleobase transmembrane transporter activity, and the BP term nucleobase transport were enriched, indicating vitamin transport and/or the transport of nitrogenous bases from one side of the membrane to the other are imperative to high PE. The enrichment of these terms also identified *SLC23A2*, mentioned previously, as a candidate gene for high PE. The fourth enriched term was the cellular component term extracellular exosome, which describes gene products that localize to vesicles that are released from cells into the extracellular region via exocytosis [48]. Exosomes are involved in cell to cell communication and modulate intercellular communication at the maternal-fetal interface in pigs [55]. Therefore, the enrichment of this term suggests exosomes play a role in the cell to cell communication required for the increased efficiency of high PE placentas. Additionally, extracellular exosome was a term of 20 upregulated genes in high PE compared to low PE placentas, validating the association with high PE.

### Fetal/utero-placental measurements and differentially expressed genes

The correlation analysis performed between fetal/utero-placental measures and the DEG identified significant correlations between 8 of the DEG and placental weight or PE. GRINL1A complex locus 1 (*GCOM1*), gene 19,656 (LOC100739517), ATP-binding cassette sub-family G member 2 (*ABCG2*), and TOX high mobility group box family member 3 (*TOX3*) were positively correlated with placental weight and downregulated in high PE compared to low PE placentas. The MF of GCOM1 in pigs is unknown. The gene *ABCG2* encodes for an active transporter that is expressed in the human placenta and transports xenobiotic compounds [56, 57]. Cholesterol activity was also a GO term of *ABCG2.* The protein product of *TOX3* may be involved in chromatin remodeling, and the bending and unwinding of DNA [57]. Molecular function GO terms included chromatin binding, phosphoprotein binding, protein homodimerization activity, and estrogen response element binding. Conversely, ras-related protein rab-6B (*RAB6B*) was negatively correlated with placental weight and was upregulated in high PE compared to low PE placentas. Molecular function GO terms of *RAB6B* included GTP binding, GTPase activity, and myosin v binding. The protein encoded by *RAB6B* localizes to the golgi apparatus and may function in retrograde membrane traffic [58]. Although the function(s) of these genes within the pig placenta are largely unknown, the identification of strong correlations with placental weight, in combination with gene expression, indicates *GCOM1*, *ABCG2*, *TOX3*, and *RAB6B* may regulate the reduced placental size of high PE placentas.

Gene 12188 (LOC100156118), transmembrane protein 199 (*TMEM199*), and proto-cadherin beta 1 (*PCDHB1*) were positively correlated with PE and upregulated in high PE compared to low PE placentas. Gene 12188 encodes an uncharacterized protein in swine. The MF of *TMEM199* in pigs in unknown, but the protein encoded by this gene in humans may be involved in golgi homeostasis [57]. The gene *PCDHB1* was among the 10 most upregulated genes. The specific function of PCDHB1 is unknown, but PCDHB1 may be a calcium dependent cell to cell adhesion protein [57]. Given the strong positive correlations of gene 12188, *TMEM199*, and *PCDHB1* with PE, further research is warranted to determine the specific functions these genes may have within the pig placenta.

## Conclusion

Placental efficiency, quantified by the ratio of fetal weight to placental weight, was determined within maternal line gilt litters to compare expression profiles of high PE feto-placental units to low PE feto-placental units. Mean fetal weight was not significantly different between the high PE group and low PE group, but placental weight was significantly reduced in in the high PE group, verifying comparisons were of similarly sized pigs grown on different sized placentas. Likewise, the absence of significant differences in fetal measures indicated any negative effects of a reduced placental size on fetal growth were not evident by day 95 of gestation. The comparison of gene expression profiles in the placenta and adjacent endometrium of high PE and low PE feto-placental units identified 214 DEG in the placenta and no DEG in the endometrium, confirming that the placenta responds to the fetus.

Gene ontology functional classification analysis of the 103 upregulated and 111 downregulated genes identified common MF and BP. The MF with the greatest representation among the DEG were catalytic activity, binding, and transporter activity. The BP with the greatest representation among the DEG were metabolism, biological regulation, and localization. Further investigation into the candidate genes associated with these terms partially supported the hypothesis and suggested extremes of PE are differentially regulated, affecting components of placental transport capacity like nutrient transport and blood flow. Conversely, DEG with growth factor activity were minimal and alternative functions were identified, indicating the complexity of the relationship between placental and fetal weights.

Overall, the results of this study support the use of PE as a marker of placental function and provide new insights into compensatory mechanisms that enable comparable fetal growth despite a reduced placental size. In swine, PE may provide an opportunity to optimize reproductive performance by normalizing the reduced birth weights of larger litters and in turn increasing pre-weaning survival; however, further research is required to effectively incorporate PE into selection schemes. Identifying associations between the phenotype and genome may be useful. Additionally, a limitation of this study was the method used to define high and low PE. Using the highest and lowest PE value within a litter is both advantageous and restrictive as it ensures comparisons are only between the very most and least efficient units, but excludes feto-placental units with efficiencies that are closer to the average. Therefore, it is suggested that future research determine the most appropriate method of defining high and low PE. Lastly, the role extracellular exosomes play in PE and the impact of environmental effects on PE is also of interest.

## Methods

### Animal management

All procedures were approved by the West Virginia University Animal Care and Use Committee (WVU-ACUC; ACUC # 10–0505). Eight Camborough 23 gilts (experimental unit; *N* = 8), owned by the WVU Animal Science Farm (Morgantown, WV), were group housed in a hoop structure equipped with tunnel ventilation and inspected annually by WVU-ACUC. Gilts were monitored for estrous behavior beginning at 5 months of age. The first estrus was observed and recorded. Gilts were bred by artificial insemination 12 and 24 h after the onset of a second estrus (6–7 months of age) using Pig Improvement Company (PIC) 1025 pooled maternal line semen (Birchwood Genetics, West Manchester, OH). Assignments for gestational day 95 ovario-hysterectomies (113–114 average day of farrowing) were randomly assigned at the time of breeding. Following breeding, gilts remained in this structure and were group housed throughout gestation.

### Surgical procedure

At least 2 days before surgery, gilts were moved to the Food Animal Research Facility at the WVU Animal Science Farm. Gilts were taken off of feed 12 h before surgery. On the morning of the day of surgery, gilts (205.63 ± 37.6 kg) were anesthetized via jugular venipuncture using ketamine (3 mg/kg) and xylazine (2 mg/kg). Atropine sulfate (0.05 mg/kg) was administered to reduce salivation and isoflurane was used to maintain anesthesia.

Gilts were placed in dorsal recumbency and a mid-ventral incision was made to expose the gravid uterus. An antimesometrial incision was then made to open the uterus and expose the feto-placental units. Two tags were attached to the umbilical cord of each feto-placental unit, identifying which uterine horn the fetus (observational unit) originated from and the location within that uterine horn. The umbilical cord was cut between the two tags to ensure a tag remained with the placenta and the fetus. All fetuses were removed, and then the uterus was removed from the dam and set aside for processing. Sodium pentobarbital (400 mg/mL, Sigma Aldrich, St. Louis, MO) was used to euthanize the gilt.

### Fetal and utero-placental measurements

Fetal weight, CRL, and girth were recorded as fetuses were removed from the uterus. Fetal necropsies were performed to obtain heart weight, liver weight, brain weight, and left hindquarter ST weight. The uterus was opened along the antimesometrial side and laid flat. Boundaries of each placenta were identified and a sample (~ 6.5 cm^2^) of all tissue layers (placenta, endometrium, and myometrium) was collected from an area void of calcium deposits and representative of the entire placenta. Samples were placed in tissue cassettes and fixed in neutral buffered formalin for histological processing. Then each placenta was peeled away from the endometrium and weighed. At this time representative samples, as described by Krombeen and others [5], were taken from both the placenta and the adjacent endometrium, placed in 2.0 mL cryovials (filled to 1.8 mL), and snap frozen in liquid nitrogen for RNA extraction. Implantation site length for each placenta was measured in the empty uterus using avascular bands as boundaries. Placental efficiency was determined for each feto-placental unit by dividing fetal weight by placental weight.

Similar to Krombeen and others [5], tissue cross sections containing placenta, endometrium, and myometrium were fixed in formalin, dehydrated with graded ethanol and xylenes, perfused with molten paraffin, and embedded in paraffin molds. Five micrometer sections were fixed to glass slides. Two sections for each fetus were stained using periodic acid and Schiff’s reagent (Sigma Aldrich, St. Louis, MO). Along the placental-endometrial interface, two fields per section were visualized (Nikon Eclipse TE2000–5, Nikon Instruments Inc., Melville, NY) and captured (Retiga 2000R, Q Imaging, Surrey, BC, Canada; Q Capture, Quantitative Imaging Corporation, v2.90.1, Surrey, BC, Canada) for a total of four fields visualized. Northern eclipse v6.0 software (Empix Inc., North Tonawanda, NY) was used to analyze images. Placental and endometrial tissues were outlined separately, and then total number of vessels, total area of vessels, and total area selected were measured. Vascular density was determined by dividing the area of the vessels by the total area selected. Replicates were averaged.

To compare fetal and utero-placental measurements of high PE and low PE units, the feto-placental unit with the highest PE and the feto-placental unit with the lowest PE in each litter (*n* = 8) were selected, creating the high PE group (*n* = 8, PE range 3.96 to 7.84) and the low PE group (*n* = 8, PE range 1.92 to 3.19) used for analysis. Statistical analyses were conducted using JMP Pro version 12.2.0 (SAS Institute Inc., Cary, NC 1989–2007). A linear mixed effects model was used to analyze each dependent variable (placental wt, fetal wt, ISL, CRL, girth, heart wt, liver wt, brain wt, ST wt, placental VD, and endometrial VD), with PE, sex, and PE*sex as fixed effects, and a random effect to account for pigs nested within dam. A significance level of 0.05 was used for all statistical tests.

### Differential gene expression and gene ontology

Endometrial and placental samples from the most efficient (2 female, 6 male) and least efficient (3 female, 5 male) feto-placental unit in each litter (*n* = 8) were processed at the Clemson University Genomics & Computational Laboratory (CU-GCL). Total RNA was extracted from the endometrial (*n* = 16) and placental (*n* = 16) samples in duplicate using a RNeasy Plus Universal Mini Kit (Qiagen, Valencia, CA) and all extractions were performed according to the manufacturer’s instructions. An aliquot of each sample was qualitatively assayed for purity using UV spectroscopy via the Nanodrop8000 (ThermoFisher Scientific, Waltham, MA) to determine the 260/280 and 260/230 ratios, respectively. RNA integrity was measured using an Agilent 2100 Bioanalyzer (Agilent Technologies, Santa Clara, CA, USA). All RNA purity ratios, were > 1.8 and all RNA integrity numbers (RIN) were approximately 6. Total RNA was quantitated with the Broad Range Assay in the Qubit (ThermoFisher Scientific, Waltham, MA).

Each sample was normalized to a standard input concentration of 2 μg for sequencing library preparation. Stranded mRNA sequencing libraries were prepared manually at the CU-GCL with the TruSeq Stranded mRNA kit (Illumina, San Diego, CA) following the manufacturer’s recommended procedures. Sequencing data was collected on the HiSeq2500 (Illumina, San Diego, CA) using v4.0 chemistry and 2x125bp paired-end reads. Post sequencing, raw sequence reads were transferred to Clemson University’s Palmetto Cluster for analysis.

Sequence reads were quality validated with the FastQC software [59], followed by read preprocessing to remove adapter and primer sequences with the Trimmomatic software [60]. Processed sequence reads were aligned to the v10.2 *Sus Scrofus* reference genome assembly [61] with the GSNAP read alignment tool [62]. Sorted and indexed. BAM files were prepared from the. SAM output of GSNAP using Samtools [63]. Uniquely mapped read abundance per gene was determined with the featureCounts software in reversely stranded mode [64], and the count data per sample was output and transformed to tabular format.

Relative pairwise changes in gene level expression were determined with the edgeR software package [65]. Transcriptome comparisons were made using a generalized linear model and pairwise comparisons were made to compare low efficiency versus high efficiency conditions in a tissue specific manner. Differentially expressed genes were determined and filtered for significance using the FDR of 0.10 [66, 67]. Gene level fold-change values were output in tabular format and genes abounding thresholds were listed as candidate genes.

Candidate gene lists containing upregulated and downregulated genes in the placenta were functionally classified using the Panther database version 14.0 [68, 69] and AmiGO 2 version 2.5.12 was utilized to search GO term definitions [48, 70, 71]. Hierarchical functional classification was used to categorize genes according to the activity of the gene product (molecular function) and the pathway or processes the gene product functions in (biological process) [70, 71]. Gene ontology slim terms were utilized to classify gene lists according to defined terms. Percentages equal the number of genes within the input list with that MF or BP divided by the total number of MF or BP in the input list (gene hits against total number of function hits or gene hits against total number of process hits). Candidate gene lists containing upregulated and downregulated genes in the placenta were also independently tested for statistical enrichment (FDR < 0.05) with the GOSeq software tool [72].

The subset of candidate genes listed in Table 2 were associated with the MF and BP with the greatest representation in the DEG and were selected based on log_2_FC (10 most upregulated or downregulated genes) and/or GO terms related to nutrient transport, angiogenic activity, or growth factor activity.

### Fetal/utero-placental measurements and differentially expressed genes

To identify relationships between the following measurements: fetal weight, placental weight, PE, CRL, brain weight, ST weight, ISL, placental VD, and endometrial VD, and the 214 DEG in high PE compared to low PE placentas, Pearson’s correlation coefficient (*r*) was estimated using the cor function in R [73]. A FDR adjustment was applied to correct for multiple comparisons using the p.adjust function in R. A significance level of 0.05 was used to identify significant correlations.

## Abbreviations

*ABCB1*: ATP-binding cassette subfamily B member 1 isoform X2
*ABCG2*: ATP-binding cassette sub-family G member 2
*AGR2*: Anterior gradient protein 2 homolog
*AK2*: Adenylate kinase 2
*ASIC1*: Acid sensing ion channel 1 isoform X2
*ATP13A3*: Probable cation-transporting ATPase 13A3
*CADM4*: Cell adhesion molecule 4
*CELA1*: Chymotrypsin-like elastase family member 1
*COCH*: Coagulation factor c homolog
CRL: Crown-rump length
*CTSH*: Pro-cathepsin H
*CYP4F22*: Cytochrome P450 family 4 subfamily f member 22
DEG: Differentially expressed genes
*DKK1*: Dickkopf-1
*EFHD1*: EF-hand domain-containing protein D1 isoform X2
*EMB*: Embigin
*ENTPD6*: Ectonucleoside triphosphate diphosphohydrolase 6
*FBP2*: Fructose-1, 6-bisphosphatase isoenzyme 2
FDR: False discovery rate adjusted *p*-value
*GCA*: Grancalcin
*GCOM1*: Myocardial zonula adherens protein
*GLA*: Alpha-galactosidase A
GO: Gene ontology
*HSPE1*: 10 KDa heat shock protein mitochondrial
ISL: Implantation site length
*ITIH3*: Inter-alpha-trypsin inhibitor heavy chain H3
IUGR: Intrauterine growth restriction
*KCNJ2*: Potassium channel inwardly rectifying subfamily J member 2
*KRTAP8–1*: Keratin associated protein 8–1
*LEP*: Leptin
LOC102159690: Solute carrier family 23 member 2-like
log_2_FC: Log_2_ fold change
*MCOLN3*: Mucolipin 3 isoform X2
*MGST3*: Microsomal glutathione S-transferase 3
*MORN5*: Morn repeat containing protein 5 isoform X8
*MRP4* or *ABCC4*: Multidrug resistance-associated protein 4-like
*PCDHB1*: Protocadherin beta-1 isoform X2
*PDHB*: Pyruvate dehydrogenase E1 component subunit beta
PE: Placental efficiency
*PEBP*: Phosphatidylethanolamine-binding protein 1
*PI16*: Peptidase inhibitor 16 precursor
PIC: Pig improvement company
*PPA1*: Inorganic pyrophosphatase
PW: Placental weight
*RAB6B*: Ras-related protein rab-6b
RIN: Rna integrity number
*RPL15*: Ribosomal protein L15
*SAA2*: Serum amyloid A2
*SARDH*: Sarcosine dehydrogenase
*SLC23A2*: Solute carrier family 23 member 2
*SLC45A3*: Solute carrier family 45 member 3
*SLC4A7*: Solute carrier family 4 member 7
*SLC52A3*: Solute carrier family 52 member 3
*SLITRK5*: SLIT and NTRK-like protein 5
*SPHKAP*: Sphingosine kinase type 1
ST: Semitendinosus
*STEAP1*: Six-transmembrane epithelial antigen of prostate 1
*STEAP2*: Six transmembrane epithelial antigen of the prostate 2
*TMEM199*: Transmembrane protein 199
*TMEM72*: Transmembrane protein 72-like isoform X2
*TOX3*: Tox high mobility group box family member 3
*TXN*: Thioredoxin
*TXNDC8*: Thioredoxin domain-containing protein 8
VD: Vascular density
WVU: West virginia university
